# Supplementary material for: Causal Relationship between Aging and Anorexia Nervosa: A White-Matter-Microstructure-Mediated Mendelian Randomization Analysis
Source: Biomedicines. 2024 Aug 16;12(8):1874. doi: 10.3390/biomedicines12081874 (PMC11351342; doi:10.3390/biomedicines12081874)
Supplement: Supplementary file 1 [file biomedicines-12-01874-s001.zip › Supplementary Figure/Figure S8/Leaveoneout plot of MR analysis for TL to ubm-b-1491.pdf]

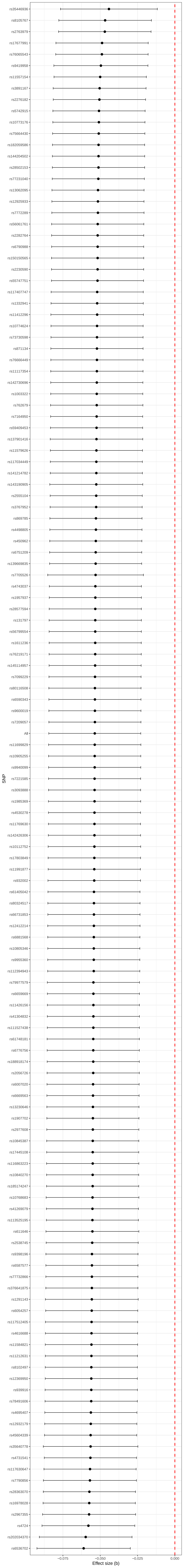
rs35446936
rs8105767
rs2763979
rs17677991
rs76065543
rs9419958
rs11557154
rs3891167
rs2276182
rs5742915
rs10773176
rs75664430
rs182059586
rs144204502
rs28502153
rs77231040
rs13062095
rs12925933
rs7772289
rs56061761
rs2282764
rs6790988
rs150150565
rs2230590
rs55747751
rs117407747
rs1332941
rs11412296
rs10774624
rs73730598
rs871134
rs76666449
rs11117354
rs142730696
rs1003322
rs762679
rs7164950
rs59409453
rs137901416
rs11579626
rs117034449
rs141214782
rs143190905
rs2555104
rs3767952
rs869785
rs4498805
rs450962
rs6751209
rs139669835
rs7705526
rs4743037
rs1957937
rs28577594
rs131797
rs56799554
rs1611236
rs76219171
rs145114957
rs7099229
rs80116508
rs6590343
rs9600019
rs7209057
All
rs11699829
rs10905255
rs9940099
rs7221585
rs3093888
rs1985369
rs4530278
rs11769630
rs142426306
rs10112752
rs17803849
rs11991877
rs932002
rs61405042
rs80324517
rs66731853
rs12412214
rs6881568
rs10805346
rs9955360
rs112394943
rs79977579
rs6659669
rs11426156
rs41304832
rs111527438
rs61748181
rs6776756
rs188918174
rs2056726
rs6007020
rs6669563
rs13230646
rs1907702
rs2977608
rs10845387
rs17445108
rs116863223
rs10840270
rs185174247
rs10768683
rs41269079
rs113525195
rs611646
rs2538745
rs9398196
rs6587577
rs77732866
rs376641875
rs1291143
rs6054257
rs117512405
rs4616688
rs11584821
rs11212631
rs8102497
rs12369950
rs939916
rs78491606
rs4695407
rs12932179
rs45604339
rs35640778
rs4731541
rs117630647
rs7790856
rs28363070
rs16978028
rs2967355
rs4724
rs202034370
rs6536702
SNP
−0.075
−0.050
−0.025
0.000
Effect size (b)
